# Association between the triglyceride-glucose index and hyperuricemia: potential role of obesity in patients with Type 2 diabetes mellitus

**DOI:** 10.3389/fendo.2025.1637543

**Published:** 2025-10-02

**Authors:** Dou Tang, Xi Gu, Yan Xuan, Fanfan Zhu, Ying Shen, Leiqun Lu

**Affiliations:** Department of Endocrinology, RuiJin Hospital Lu Wan Branch, Shanghai Jiaotong University School of Medicine, Shanghai, China

**Keywords:** the TyG index, hyperuricemia, BMI, mediation analysis, type 2 diabetes mellitus

## Abstract

**Background:**

The triglyceride-glucose (TyG) index has recently emerged as a simple surrogate marker of insulin resistance. However, the relationships among the TyG index, obesity, and hyperuricemia in individuals with T2DM remain unclear. This study investigates the associations of the TyG index and obesity with hyperuricemia in T2DM, and the possible role of obesity in these associations.

**Methods:**

In this cross-sectional study, 1,159 adults with T2DM were enrolled. The TyG index was calculated as ln [fasting triglyceride (mg/dl) × fasting plasma glucose (mg/dl)/2]. Participants were stratified into hyperuricemia and non-hyperuricemia groups based on serum uric acid levels. Multivariable logistic regression and subgroup analyses were performed to assess the association between the TyG index and hyperuricemia. Mediation analysis quantified BMI’s contribution to this relationship.

**Results:**

The prevalence of hyperuricemia was 30.7%. After adjustment for age, gender, HbA1c, diabetes duration, eGFR, HDL-C, LDL-C, hypertension, hyperlipidemia, coronary heart disease, smoking status, and alcohol consumption, each unit increase in the TyG index was independently associated with a 36% elevated risk of hyperuricemia (OR = 1.36, 95% CI: 1.10-1.68). Subgroup analyses showed consistent associations across different patient subgroups. Mediation analysis indicated that BMI accounted for 20.0% of the relationship.

**Conclusions:**

The TyG index and BMI were both associated with hyperuricemia in Chinese patients with T2DM, with BMI potentially representing an indirect link.

## 1 Introduction

Hyperuricemia has emerged as a crucial public health challenge globally. Hyperuricemia is defined by elevated serum uric acid levels, primarily due to excessive production, impaired urinary excretion, or a combination of both (1). Recent meta-analyses have demonstrated a consistent rise in hyperuricemia prevalence across China over recent decades (2). A national survey conducted in 2018–19 found that about 14.0% of adults in China have hyperuricemia (3). Similarly, in the general U.S. population, approximately 20% had hyperuricemia according to the National Health and Nutrition Examination Survey from 1999 to 2018 (4). Numerous epidemiologic studies have shown that hyperuricemia correlates with type 2 diabetes mellitus (T2DM), insulin resistance, metabolic syndrome, renal disease, cardiovascular disease, and all-cause mortality (5–8). Notably, individuals with T2DM exhibit a significantly higher prevalence of hyperuricemia compared to the general population (9–11). Therefore, identifying modifiable risk factors for hyperuricemia is critical to enable targeted prevention and management approaches.

The TyG index is a simple, cost-effective, and reliable surrogate marker of insulin resistance (IR), avoiding the technical and financial constraints linked to the hyperinsulinemic-euglycemic clamp (HEC) or HOMA-IR in regular practice (12). Accumulating evidence underscores its strong predictive value for IR-related metabolic disturbances, with studies demonstrating significant correlations between elevated TyG index and incident T2DM, cardiovascular events, chronic kidney disease, and non-alcoholic fatty liver disease (13, 14). The TyG index, derived from fasting triglyceride and glucose levels, indicates lipid and glucose metabolism dysregulation, which is mechanistically linked to hyperuricemia pathogenesis (15, 16).

Some studies have consistently demonstrated a significant association between IR and hyperuricemia (12). In the Chinese general population, a linear positive association between the TyG index and hyperuricemia has been observed (17). In contrast, a cross-sectional study from the general population in the United States revealed a non-linear, reverse U-shaped relationship between the TyG index and hyperuricemia (4). Among hypertensive individuals, this relationship remains positively correlated (18, 19). Although insulin resistance is recognized as a central pathophysiological mechanism in T2DM, evidence investigating the specific interplay between the TyG index and hyperuricemia in diabetic populations remains limited.

BMI, an indicator of obesity, is strongly associated with hyperuricemia (20–22). Existing research also shows a positive correlation between the TyG index and BMI (23). However, the complex relationship among these three factors has not been thoroughly explored, particularly in individuals with type 2 diabetes, who represent a population characterized by high insulin resistance, high incidence of hyperuricemia, and a strong association with obesity. Therefore, this study aims to examine the association between the TyG index and hyperuricemia in individuals with T2DM and to assess the potential mediating role of obesity, measured by BMI, in this relationship.

## 2 Methods

### 2.1 Study participants

A total of 1,193 consecutive T2DM inpatients at the Department of Endocrinology, RuiJin Hospital Lu Wan Branch, from March 2020 to December 2024, were retrospectively selected as the research subjects. T2DM diagnosis was established according to the 2020 American Diabetes Association (ADA) criteria [23]. Participants were excluded based on the following criteria: missing data of uric acid (n=12), missing data of TyG index (n=32), or age < 18 years (n=1). In total, 34 individuals (2.9%) were excluded, leaving 1,159 participants in the final analysis. According to the Helsinki Declaration, the study protocol was approved by the Ethics Committee of RuiJin Hospital Lu Wan Branch, Shanghai Jiaotong University School of Medicine. All participants gave their written informed consent.

### 2.2 Clinical and biochemical analysis

All participants completed structured interviews by trained research staff, including sex, age, duration of diabetes mellitus, smoking status (never, former, or current), smoking (never or current), and medical history. BMI was calculated as body weight (kg)/height (m^2^). An electronic sphygmomanometer measured SBP and DBP after a 5-minute rest period. Blood samples were collected from subjects after an overnight fast of at least eight hours, typically in the morning, ensuring standardized fasting conditions. Fasting blood glucose (FBG), glycated hemoglobin A1c (HbA1c), triglycerides (TG), total cholesterol (TC), high-density lipoprotein cholesterol (HDL-C), low-density lipoprotein cholesterol (LDL-C), uric acid (UA), and renal function tests were measured using standard methods. The estimated glomerular filtration rate (eGFR) was calculated using the abbreviated Modification of Diet in Renal Disease (MDRD) formula (24).

### 2.3 Definitions of the exposure and outcome variables

The TyG index was calculated using the formula: TyG = ln [TG (mg/dL) × FBG (mg/dL) / 2] (25). Hyperuricemia was defined as a serum uric acid level ≥ 420 μmol/L in males or ≥ 360 μmol/L in females (26).

### 2.4 Statistical analysis

Continuous variables with normal distributions (assessed by Kolmogorov-Smirnov tests and Q-Q plots) were expressed as mean ± standard deviation, while non-normally distributed variables were reported as median (interquartile range). Categorical variables were presented as frequencies (percentages). Differences between the hyperuricemia and non-hyperuricemia groups were assessed using the Student’s t-test for normally distributed continuous variables, the Mann-Whitney U test for non-normally distributed continuous variables, and the chi-square test for categorical variables. P-values for baseline comparisons are presented for descriptive purposes only and were not adjusted for multiple comparisons.

The associations between the TyG index and hyperuricemia were assessed using univariate and multivariable logistic regression models across three different models. The results are presented as odds ratios (ORs) with corresponding 95% confidence intervals (CIs). Model 1 was unadjusted. Model 2 was adjusted for age and gender. Model 3 was additionally adjusted for HbA1c, diabetes duration, estimated glomerular filtration rate (eGFR), HDL-C, LDL-C, hypertension, hyperlipidemia, coronary heart disease, smoking status, and alcohol consumption. Covariate selection was based on clinical relevance, existing literature, or a ≥ 10% change in the effect estimate when added to the model (6). In addition, restricted cubic spline (RCS) regression was performed with 3 knots at the 10th, 50th, 90th percentiles to assess potential nonlinear relationship between the TyG index and hyperuricemia after adjusting variables in Model 3.

Stratified analyses were performed to investigate the relationship between the TyG index and hyperuricemia and their interaction in different subgroups, with adjustments for corresponding confounding factors. Mediation analysis was used to evaluate whether BMI is associated with the link between the TyG index and hyperuricemia. Three distinct statistical models were employed to evaluate the adjusted mediation effect. Mediation analysis was performed using the bootstrap method with 1,000 resamples to estimate total, direct, and indirect effects and to calculate the proportion mediated (PM). A mediation effect was considered statistically significant if the 95% CI of the β coefficient did not include zero (27). Given the cross-sectional design of this study, causal relationships inferred from the mediation analysis should be interpreted with caution. Statistical significance was defined as a two-tailed P-value <0.05. All analyses were performed using SPSS software (Version 25.0), EmpowerStats, and statistical package R (Version 4.2.0). Mediation analysis was conducted in R using the mediation package (28).

## 3 Results

### 3.1 Participants’ characteristics by hyperuricemia

The characteristics of the participants are presented in **Table 1**. A total of 1159 patients with T2DM (701 males and 458 females) were enrolled in the final study, among whom 356 patients (30.7%) were diagnosed with hyperuricemia. Compared to patients without hyperuricemia, those with hyperuricemia were more likely to be younger (P = 0.003). However, no significant gender difference was observed (P = 0.062). Patients with hyperuricemia exhibited higher weight, BMI, elevated triglyceride, total cholesterol levels, along with reduced high-density lipoprotein cholesterol (all P<0.001). Additionally, they had lower estimated glomerular filtration rates (eGFR), markedly elevated serum uric acid (457.76 ± 65.90 vs. 309.47 ± 57.86 μmol/L; *P* < 0.001), higher prevalence of hypertension and hyperlipidemia, and longer diabetes duration (all *P* < 0.05). The TyG index was also significantly higher in patients with hyperuricemia than in those without (9.37 ± 0.77 vs. 9.06 ± 0.75, P<0.001). However, no significant differences were found in height, systolic blood pressure, diastolic blood pressure, fasting blood glucose, HbA1c, LDL-C, coronary heart disease status, smoking status, or alcohol consumption between groups.

**Table 1:** Clinical characteristics of participants by hyperuricemia.

Variables	Non-hyperuricemia	Hyperuricemia	*P* value
	(N = 803)	(N = 356)	
Age, years	59.74 ± 10.25	57.63 ± 12.31	0.003
Male, n (%)	500 (62.27%)	201 (56.46%)	0.062
Height, cm	166.42 ± 8.54	166.80 ± 8.49	0.479
Weight, kg	70.47 ± 12.88	75.64 ± 15.64	<0.001
BMI, kg/m^2^	25.34 ± 3.52	27.03 ± 4.19	<0.001
SBP, mmHg	127.63 ± 19.43	128.20 ± 19.23	0.645
DBP, mmHg	74.47 ± 10.88	75.21 ± 11.28	0.298
FBG, mmol/L	7.34 ± 2.44	7.15 ± 2.29	0.209
HbA1C, (%)	8.58 ± 2.12	8.37 ± 2.07	0.133
Triglyceride, mmol/L	1.45 (1.04-2.12)	1.94 (1.43-2.82)	<0.001
Total Cholesterol, mmol/L	4.93 ± 1.30	5.28 ± 1.53	<0.001
HDL-C, mmol/L	1.17 ± 0.30	1.10 ± 0.25	<0.001
LDL-C, mmol/L	3.16 ± 0.94	3.38 ± 0.99	<0.001
eGFR, mL/min/1.73m²	109.58 ± 27.90	99.94 ± 27.60	<0.001
Creatinine, umol/L	65.82 ± 17.38	71.29 ± 19.10	<0.001
Uric acid, umol/L	309.47 ± 57.86	457.76 ± 65.90	<0.001
The TyG index	9.06 ± 0.75	9.37 ± 0.77	<0.001
Hypertension, n (%)	426 (53.05%)	215 (60.39%)	0.02
Hyperlipidemia, n (%)	271 (33.75%)	156 (43.94%)	<0.001
Coronary Heart Disease, n (%)	93 (11.58%)	47 (13.20%)	0.435
Diabetic duration, years	9.73 ± 8.59	8.45 ± 8.36	0.018
Smoking status, n (%)			0.075
Never smoker	426 (53.25%)	215 (60.39%)	
Ex-smoker	96 (12.00%)	38 (10.67%)	
Current smoker	278 (34.75%)	103 (28.93%)	
Alcohol consumption, n (%)	78 (9.75%)	36 (10.11%)	0.849

Data are presented as mean ± SD, median (interquartile range), or number (%).

BMI, body mass index; SBP, systolic blood pressure; DBP, diastolic blood pressure; FBG, fasting blood glucose; HbA1C, glycosylated hemoglobin; HDL-C, high-density lipoprotein-C; LDL-C, low-density lipoprotein-C; eGFR, estimated glomerular; the TyG index, the triglyceride-glucose index.

### 3.2 Association between TyG and BMI with hyperuricemia

The univariate and multivariable regression models are shown in **Table 2**. In the unadjusted model (Model 1), each unit increase in the TyG index was associated with a 1.67-fold increase of hyperuricemia (95% CI: 1.42-1.97; P < 0.001). When the TyG index was categorized into tertiles, the odds of hyperuricemia were significantly higher in the second (OR = 2.21, 95% CI: 1.59–3.08, P < 0.001) and third tertiles (OR = 2.67, 95% CI: 1.93–3.70, P < 0.001) compared with the first tertile. The association remained significant in multivariable logistic regression models after adjusting for potential confounders. After adjusting for age, gender, HbA1c, diabetes duration, eGFR, HDL-C, LDL-C, hypertension, hyperlipidemia, coronary heart disease, smoking status, and alcohol consumption in model 3, each one-unit increase in the TyG index was associated with an OR of 1.36 (95% CI: 1.10-1.68; P = 0.005) for hyperuricemia. Similarly, the adjusted ORs were 1.64 (95% CI: 1.15–2.35; P = 0.006) for the second TyG tertile and 1.75 (95% CI: 1.19–2.56; P = 0.004) for the third tertile, compared with the first tertile (P for trend <0.01).Accordingly, the relationship between the TyG index and hyperuricemia appeared to be approximately linear in RCS (P for nonlinearity = 0.123, **Supplementary Figure S1**).

**Table 2:** Association between the triglyceride-glucose index and BMI with hyperuricemia.

Variables	Model 1		Model 2		Model 3	
	OR (95%CI)	*P* value	OR (95%CI)	*P* value	OR (95%CI)	*P* value
TyG (continuous)	1.67 (1.42, 1.97)	<0.001	1.65 (1.39, 1.96)	<0.001	1.36 (1.10, 1.68)	0.005
TyG (tertile)						
T1:6.91-8.81	Reference		Reference		Reference	
T2:8.82-9.37	2.21 (1.59, 3.08)	<0.001	2.20 (1.58, 3.06)	<0.001	1.64 (1.15, 2.35)	0.006
T3:9.38-12.28	2.67 (1.93, 3.70)	<0.001	2.53 (1.81, 3.53)	<0.001	1.75 (1.19, 2.56)	0.004
*P* for trend	<0.001		<0.001		0.006	
BMI	1.12 (1.09, 1.16)	<0.001	1.12 (1.08, 1.16)	<0.001	1.08 (1.04, 1.12)	<0.001

OR, odds ratio; CI, confidence interval; TyG, triglyceride-glucose; BMI, body mass index.

Model 1: unadjusted.

Model 2: adjusted for age and gender.

Model 3: adjusted for the variables in Model 2 plus HbA1c, the duration of diabetes, eGFR, HDL-C, LDL-C, hypertension, hyperlipidemia, coronary heart disease, smoking status, and alcohol consumption.

The correlation between BMI and hyperuricemia was also rigorously analyzed (**Table 2**). In the crude model (Model 1), each one-unit increase in BMI was associated with a 12% increased risk of hyperuricemia (OR = 1.12, 95% CI: 1.09–1.16, P<0.001). After adjusting for age and gender, Model 2 confirmed similar findings (OR = 1.12, 95% CI: 1.08–1.16, P<0.001). With further adjustment for potential confounders in Model 3, the risk slightly declined but remained significant (OR = 1.08, 95% CI: 1.04–1.12, P<0.001).

### 3.3 Association between TyG and BMI

**Table 3** presents the results of the multivariable linear regression analysis examining the association between the TyG index and BMI. In the unadjusted model (Model 1), the TyG index showed a positive association with BMI (β=1.62, 95% CI: 1.35–1.89, P < 0.001). After adjustment for age and sex in Model 2, the association remained significant (β=1.31, 95% CI: 1.03–1.58, P < 0.001). Further adjustment in model 3 for additional covariates, including HbA1c, diabetes duration, eGFR, HDL-C, LDL-C, hypertension, hyperlipidemia, coronary heart disease, smoking status, and alcohol consumption attenuated the strength of the association, but it remained statistically significant (β=0.95, 95% CI: 0.63–1.26, P < 0.001).

**Table 3:** The association between the triglyceride-glucose index and BMI.

Variables	Model 1		Model 2		Model 3	
	β (95%CI)	*P* value	β (95%CI)	*P* value	β (95%CI)	*P* value
TyG (continuous)	1.62 (1.35, 1.89)	<0.001	1.31 (1.03, 1.58)	<0.001	0.95 (0.63, 1.26)	<0.001
TyG (tertile)						
T1:6.91-8.81	Reference		Reference		Reference	
T2:8.82-9.37	1.68 (1.17, 2.20)	<0.001	1.59 (1.09, 2.09)	<0.001	1.05 (0.54, 1.55)	<0.001
T3:9.38-12.28	2.71 (2.19, 3.22)	<0.001	2.19 (1.68, 2.70)	<0.001	1.47 (0.91, 2.02)	<0.001
*P* for trend	<0.001		<0.001		<0.001	

β, regression coefficient; CI, confidence interval. TyG, triglyceride-glucose.

Model 1: unadjusted.

Model 2: adjusted for age and gender.

Model 3: adjusted for the variables in Model 2 plus HbA1c, the duration of diabetes, eGFR, HDL-C, LDL-C, hypertension, hyperlipidemia, coronary heart disease, smoking status, and alcohol consumption.

To ensure the robustness of the results, we further categorized the TyG index into tertiles. Compared with the lowest tertile, the highest tertile of the TyG index was associated with a higher BMI (β=1.47, 95% CI: 0.91–2.02, P < 0.001). Moreover, a significant dose-response relationship was observed (P for trend <0.001).

### 3.4 Subgroup analyses

**Figure 1:**
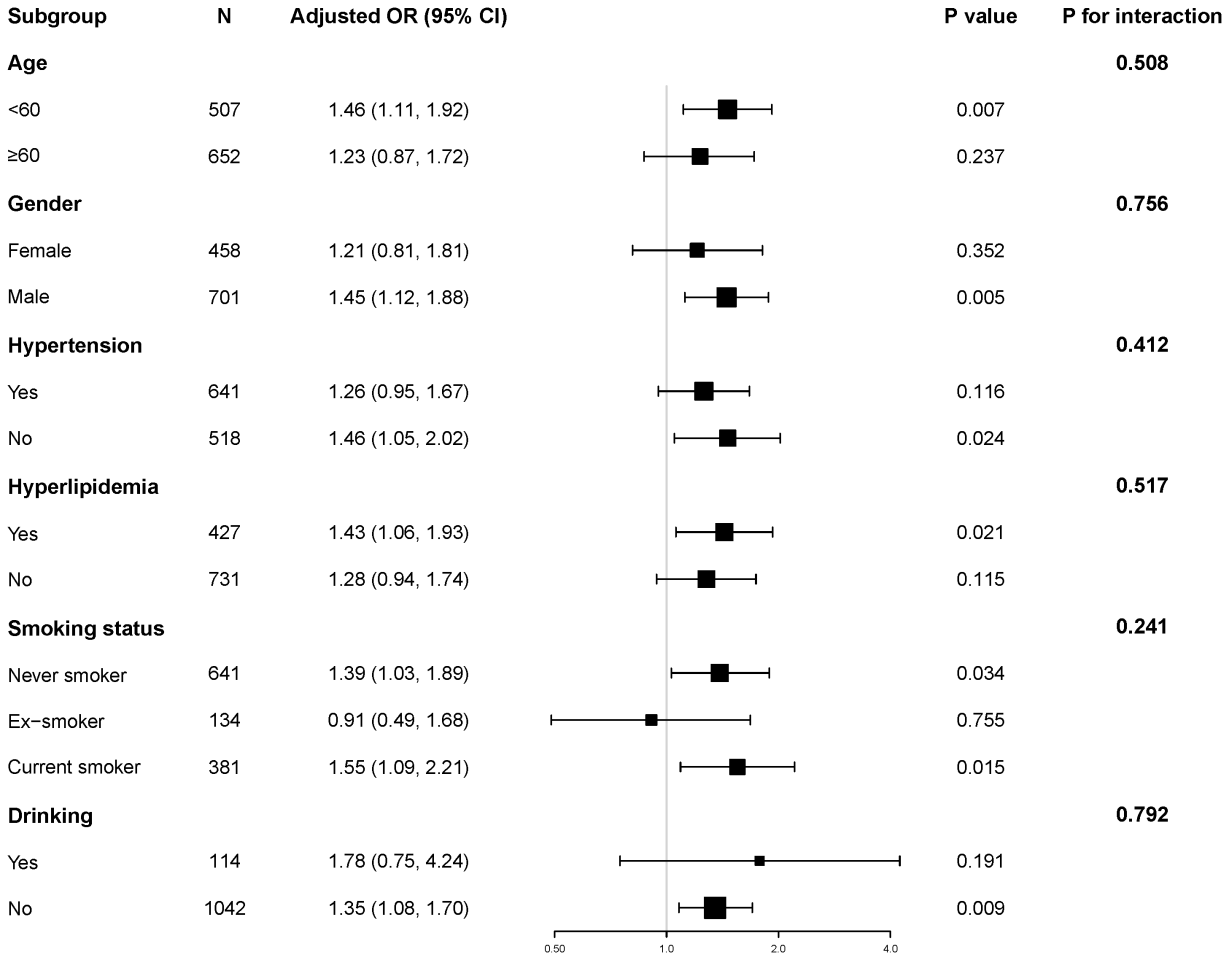
Subgroup and interaction analyses of the TyG index and hyperuricemia. Adjusted for age, gender, HbA1c, the duration of diabetes, eGFR, HDL-C, LDL-C, hypertension, hyperlipidemia, coronary heart disease, smoking status, and alcohol consumption.

Stratified analyses were performed to explore the robustness of the association between the TyG index and hyperuricemia, as shown in **Figure 1**. We found a positive association between the TyG index and hyperuricemia across subgroups defined by age, gender, hypertension status, hyperlipidemia, smoking status and alcohol consumption.

Notably, we observed no significant interaction effects among these subgroups (all P for interaction > 0.05). These results support the independent and robust correlation between TyG and hyperuricemia across all subgroups.

### 3.5 Mediation analyses

**Figure 2:**
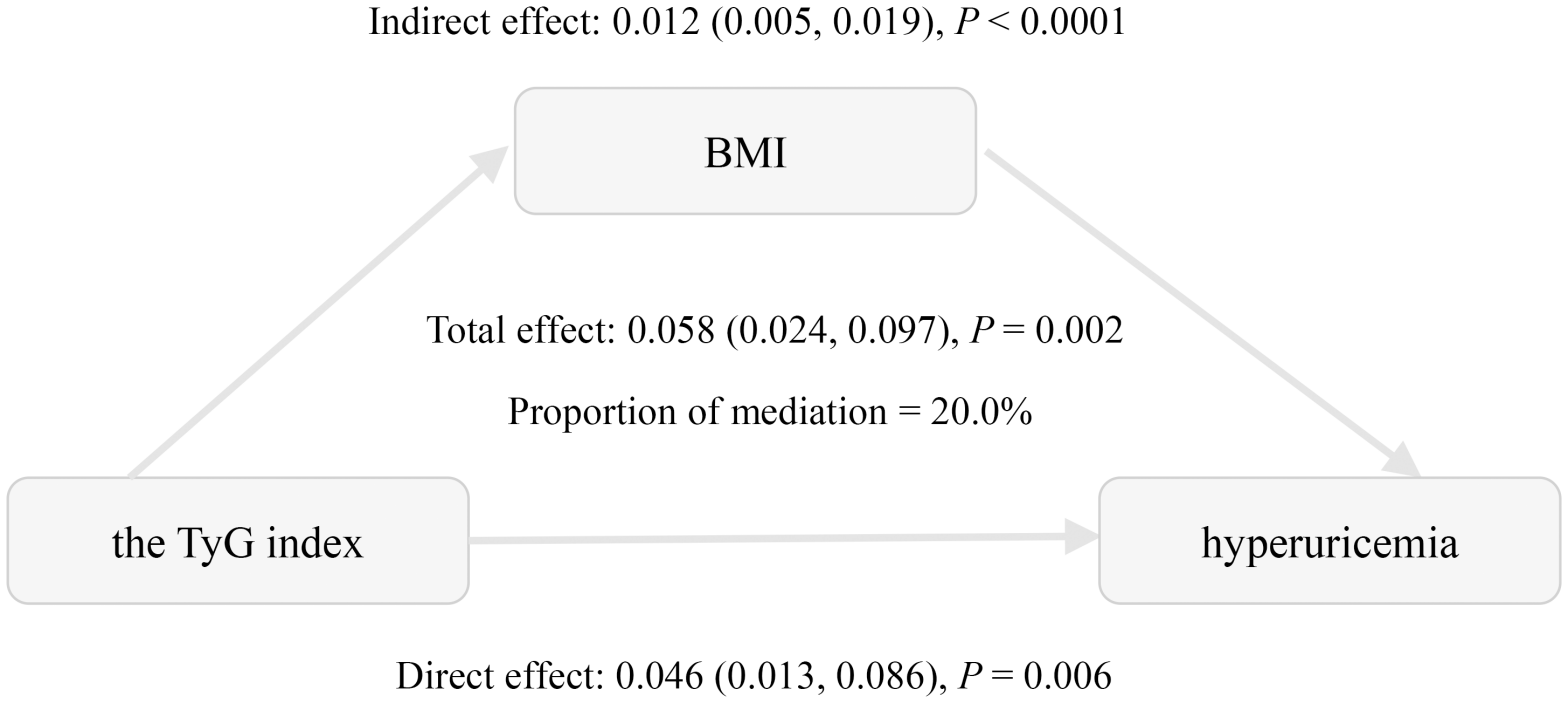
Mediation effect of BMI in the association between the triglyceride-glucose index and hyperuricemia. Adjusted for age, gender, HbA1c, the duration of diabetes, eGFR, HDL-C, LDL-C, hypertension, hyperlipidemia, coronary heart disease, smoking status, and alcohol consumption.

Mediation analysis was conducted to assess the relationship of BMI with the TyG index and hyperuricemia (**Table 4**, **Figure 2**). The total effect of the TyG index on hyperuricemia was statistically significant across all three models. The indirect effect through BMI was also significant, accounting for approximately 20.0% to 27.9% of the total effect, indicating a meaningful mediation role of BMI. Even after adjusting for multiple potential confounders, including age, gender, HbA1c, diabetes duration, eGFR, HDL-C, LDL-C, hypertension, hyperlipidemia, coronary heart disease, smoking status, and alcohol consumption, both the direct and indirect effects remained statistically significant. These findings are consistent with a potential role of BMI in the relationship between the TyG index and hyperuricemia.

**Table 4:** Mediation analysis of the association between the triglyceride-glucose index and hyperuricemia mediated by BMI.

	Model 1		Model 2		Model 3	
	Estimate (95%CI)	*P* value	Estimate (95%CI)	*P* value	Estimate (95%CI)	*P* value
Total effect	0.101 (0.072, 0.130)	<0.001	0.099 (0.068, 0.129)	<0.001	0.058 (0.024, 0.097)	0.002
Indirect effect	0.028 (0.018, 0.041)	<0.001	0.023 (0.014, 0.034)	<0.001	0.012 (0.005, 0.019)	<0.001
Direct effect	0.073 (0.042, 0.104)	<0.001	0.075 (0.043, 0.107)	<0.001	0.046 (0.013, 0.086)	0.006
PM, %	27.9%		23.6%		20.0%	
*P* value	<0.001		<0.001		0.002	

OR, odds ratio; CI, confidence interval; PM, proportion mediated.

Model 1: unadjusted.

Model 2: adjusted for age and gender.

Model 3: adjusted for the variables in Model 2 plus HbA1c, the duration of diabetes, eGFR, HDL-C, LDL-C, hypertension, hyperlipidemia, coronary heart disease, smoking status, and alcohol consumption.

## 4 Discussion

This study explored the relationships between insulin resistance, obesity, and hyperuricemia in the context of T2DM. We demonstrated significant positive associations between the TyG index, BMI, and hyperuricemia in Chinese patients with T2DM. These associations remained significant after adjusting for confounding factors. Subgroup analyses further confirmed the consistent relationship between the TyG index and hyperuricemia. Notably, mediation analysis reveals that BMI accounts for 20.0% of this association, suggesting that obesity may be involved in this relationship.

A recent systematic review and meta-analysis reported a pooled hyperuricemia prevalence of 27.28% among African individuals with T2DM, with regional variations ranging from 24.72% in North Africa to 33.72% in Central Africa (10). Similar regional differences exist in China. A previous study in Southwest China revealed a prevalence rate of 21.24% in diabetic patients (11), whereas another research in Urumqi reported a notably lower prevalence of 12.6% (29). In our research, 30.7% of individuals with type 2 diabetes were found to have hyperuricemia. These variations may reflect differences in genetic profiles, dietary habits, lifestyle choices, and environmental exposures across regions. While most epidemiological studies report higher hyperuricemia prevalence in males, our findings showed no significant sex difference. This contradicts established phenomenon where premenopausal women typically exhibit lower uric acid levels due to estrogen’s protective effect. Notably, in our study, the female participants had a median age of 63 years (interquartile range [IQR]: 57-67), suggesting that the majority were likely postmenopausal and had lost the protective effect of estrogen. Consequently, their serum uric acid levels progressively elevated, approximating those observed in males (30–32).

Accumulating evidence establishes a significant association between the TyG index and hyperuricemia. Shi et al. revealed a linear relationship between TyG and hyperuricemia in the general Chinese population, with each SD increase in TyG corresponding to a 54.1% higher risk of hyperuricemia (17). Recently, Qiu et al. further demonstrated a positive, reverse U-shaped association in U.S. adults, suggesting a complex relationship that may be influenced by geographic and ethnic factors (4). Moreover, several studies have confirmed this association in hypertensive populations (18, 19). However, evidence specific to diabetic populations remains limited (12). Consistent with prior studies, our research confirmed a positive and linear association in the Chinese diabetic population. Subgroup analyses and interaction tests further demonstrated its consistency across diverse patient subgroups. So far, the role of obesity as a mediator between the TyG index and hyperuricemia has not been thoroughly investigated. A cross-sectional analysis of the National Health and Nutrition Examination Survey (NHANES) revealed that BMI mediated 46.8% of this association in the general U.S. population (33). In contrast, a study conducted among middle-aged and elderly hypertensive individuals in China found that BMI mediated only 8.9% of this association (34). Notably, our study revealed a mediation proportion of 20.0% for the association between the TyG index and hyperuricemia in patients with T2DM, a proportion intermediate between the 46.8% reported in the general U.S. population and the 8.9% observed in middle-aged and elderly Chinese hypertensive patients. These discrepancies may arise from metabolic characteristics in a specific population. Ethnic differences in susceptibility to insulin resistance may also contribute to the heterogeneity in the mediation effect of BMI across populations (35). Additionally, unmeasured confounders and methodological differences may also contribute to the observed heterogeneity. To the best of our knowledge, this is the first study to investigate the mediating effect of BMI on the association between the TyG index and hyperuricemia in a diabetic population.

The mechanisms behind this observation are not yet clearly established, but several potential biological mechanisms may account for this observation. The TyG index, derived from triglycerides and fasting blood glucose levels, reflects dysregulated lipid and glucose metabolism, both implicated in hyperuricemia pathogenesis (15, 16). Animal studies suggest IR exacerbates hyperuricemia primarily through enhancing urate reabsorption via increased expression of urate transporter 1 (URAT1) and glucose transporter 9 (GLUT9), elevating serum uric acid levels (36, 37). Obesity, as a key component of metabolic syndrome, promotes free fatty acid (FFA) release, further enhancing insulin resistance and urate transporters expression (33, 37, 38). Adipocytes secrete inflammatory factors such as TNF-α and IL-6, as well as adipokines like leptin, adiponectin, and resistin. These substances contribute to insulin resistance and induce a chronic low-grade inflammatory state, thereby damaging the kidneys (39, 40). This may ultimately impair uric acid excretion and elevate uric acid levels.

The study has several limitations. Firstly, because TyG, BMI, and SUA were measured simultaneously, temporality cannot be established and causal inference is not possible. Our mediation analysis revealed indirect associations consistent with a potential pathway from TyG through BMI to hyperuricemia, but alternative or reverse pathways, such as hyperuricemia leading to insulin resistance (41), are biologically plausible. These findings are exploratory and hypothesis-generating, and require confirmation in future longitudinal or interventional studies. Secondly, residual and unmeasured confounding may exist, including dietary intake, certain medications, and renal factors beyond eGFR, and potential bias from self-reported variables such as smoking, alcohol consumption, and medical history. Additionally, both TyG and hyperuricemia were measured from the same blood sample, introducing a shared-source bias that may inflate the observed association. Results should be interpreted with caution. Thirdly, consecutive inpatients may represent a population with more severe or poorly controlled diabetes, and as a single-center study of Chinese patients, caution is needed when generalizing these findings.

## 5 Conclusions

In this cross-sectional study of Chinese patients with T2DM, both the TyG index and BMI were independently associated with hyperuricemia. The observed associations were consistent with a possible indirect link through BMI, but causal mediation cannot be established due to the study design. These findings suggest that obesity may partly explain the relationship between insulin resistance reflected by the TyG index and hyperuricemia. Weight management and lifestyle interventions targeting both insulin resistance and obesity may therefore have potential clinical value in reducing hyperuricemia risk in this population. Future longitudinal studies with prospective follow-up and external validation are needed to clarify whether integrating insulin resistance and obesity into multivariable models can improve risk stratification for hyperuricemia in patients with type 2 diabetes.

## Data Availability

The raw data supporting the conclusions of this article will be made available by the authors, without undue reservation.
