# Supplementary material for: Association between the triglyceride-glucose index and hyperuricemia: potential role of obesity in patients with Type 2 diabetes mellitus
Source: Front Endocrinol (Lausanne). 2025 Oct 2;16:1637543. doi: 10.3389/fendo.2025.1637543 (PMC12530394; doi:10.3389/fendo.2025.1637543)
Supplement: Supplementary file 1 [file DataSheet1.docx]

Supplementary Material

## Supplementary Figures


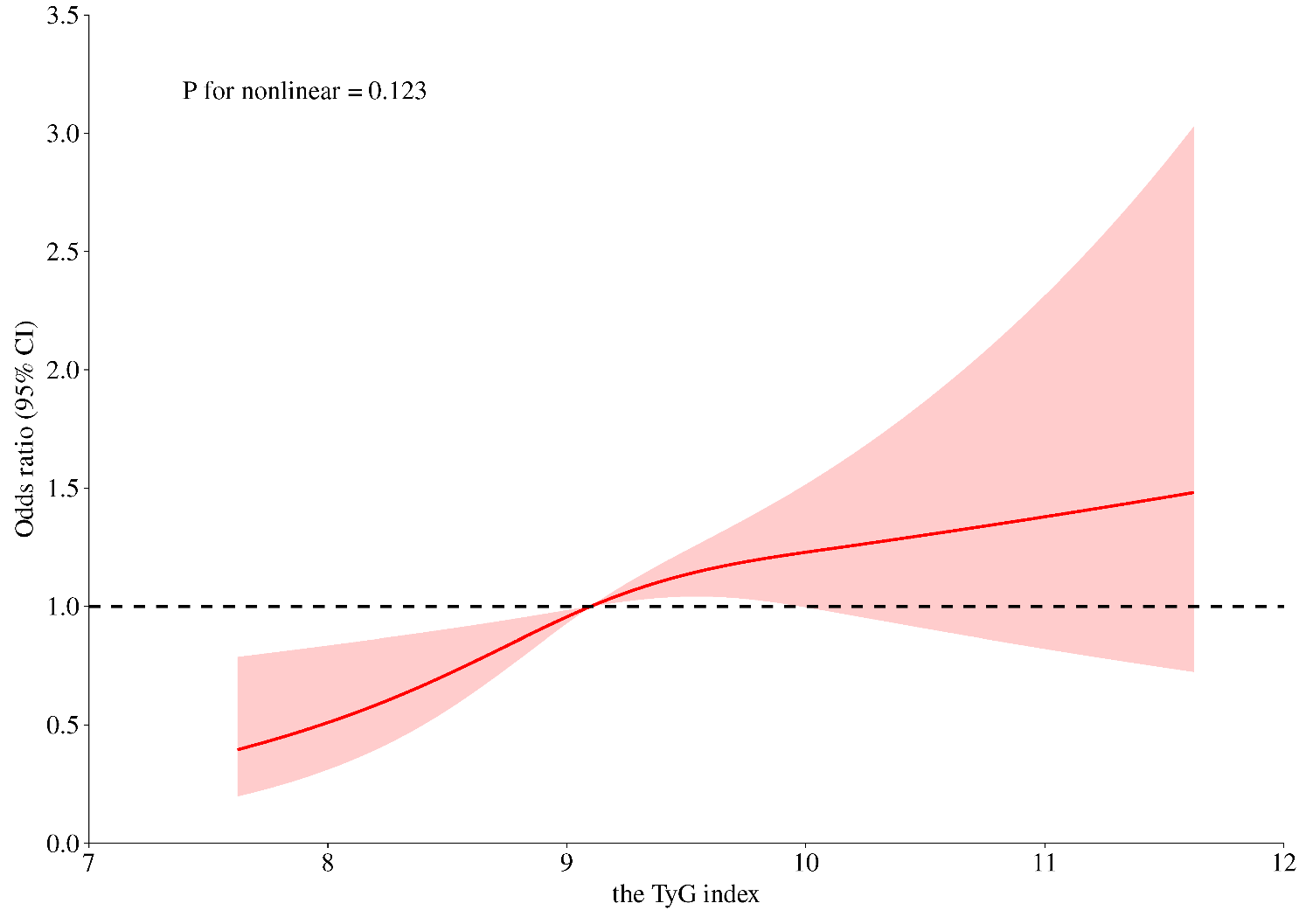


**Supplementary Figure 1.** The dose-response relationship between the TyG index and hyperuricemia. The restricted cubic spline (RCS) model was adjusted for age, sex, HbA1c, the duration of diabetes, eGFR, HDL-C, LDL-C, hypertension, hyperlipidemia, coronary heart disease, smoking status, and drinking.
